# Current status of palliative care delivery and self-reported practice in ICUs in Japan: a nationwide cross-sectional survey of physician directors

**DOI:** 10.1186/s40560-022-00605-8

**Published:** 2022-03-18

**Authors:** Yuko Igarashi, Yuta Tanaka, Kaori Ito, Mitsunori Miyashita, Satomi Kinoshita, Akane Kato, Yoshiyuki Kizawa

**Affiliations:** 1 Department of Palliative Medicine, Kobe University Graduate School of Medicine, 7-5-1 Kusunokicho, Chuo-ku, Kobe, Hyogo 650-0017 Japan; 2 Department of Palliative Nursing, Health Sciences, Tohoku University Graduate School of Medicine, 2-1 Seiryo-machi, Aoba-ku, Sendai, Miyagi 980-8575 Japan; 3 Department of Emergency Medicine, Division of Acute Care Surgery, Teikyo University School of Medicine, 2-11-1 Kaga, Itabasi-ku, Tokyo, 173-8606 Japan; 4 College of Nursing, Kanto Gakuin University, 1-50-1 Mutsuurahigashi, Kanazawa-ku, Yokohama Kanagawa, 236-8503 Japan; 5 Department of Adult and Geriatric Nursing, School of Health Science, Shinshu University, 3-1-1, Asahi, Matsumoto, Nagano, 390-8621 Japan

**Keywords:** Palliative care, Intensive care unit, Nation-wide survey, Life-sustaining treatments, Palliative care screening

## Abstract

**Background:**

It is currently unknown how widespread is the practice of palliative care in intensive care units (ICUs) in Japan. This study aimed to determine evaluate the delivery and self-reported practice of palliative care in ICUs in Japan.

**Methods:**

A self-administered questionnaire was sent to the physician directors of all 873 ICUs in Japan in August 2020.

**Results:**

Of the 873 institutions, 439 responded the questionnaire (response rate: 50%) and 413 responses were included in the analysis. The responding physicians thought palliative care was appropriate for physical symptoms (36%, 95% Confidence Interval [CI] 32–41), the provision of information (32%, 95% CI: 28–37), psychological distress (25%, 95% CI: 21–29) and in Post Intensive Care Syndrome (PICS) prevention (20%, 95% CI: 17–24). Only 4% (95% CI: 2–6) of participants indicated that they always provided palliative care screening for the patients admitted to the ICU. The most common method to determine eligibility for palliative care was the “prediction of prognosis by clinician’s experience” (54%, 95% CI: 50–59). Thirty-one percent (95% CI: 27–36) of participants responded that there was no clear method used to decide which patients need palliative care. Fifty-four percent of the participants answered they had no standardized protocols for symptom management at all. Less than 5% answered they had standardized protocols for end-of-life symptom management or terminal weaning off mechanical ventilation including extubation of endotracheal tubes.

**Conclusions:**

In Japan, the dissemination of palliative care and its integration into ICU care appears insufficient. To improve the quality of life of patients who are admitted to ICU, it may be useful to implement palliative care screening and multidisciplinary conferences, to develop standardized protocols for symptom management and withholding or withdrawing of life-sustaining treatment, and to educate primary palliative care for all ICU physicians.

## Background

Intensive care has developed rapidly throughout the world over the last few decades, contributing to the high life-saving rate of patients by providing life-sustaining treatment and other advanced medical care for seriously ill patients [1–3]. Nevertheless, it is not uncommon for patients admitted to an Intensive Care Unit (ICU) to die in the hospital despite receiving advanced medical care. Even if the patients survive hospital, they often require long-term intensive care or transferal to another hospital with serious disabilities. According to the 2019 Japanese Intensive Care PAtient Database (JIPAD), a practice registry of the Japanese Society of Intensive Care Medicine, 8.5% of all ICU patients died in hospital and 17.1% required hospital transfer [1]. When restricted to critically ill adult patients, the rates were 13.2% and 23.5%, respectively [1]. These data mean that at least one-third of adult critically ill patients admitted to the ICU will either die or live with severe disabilities.

The World Health Organization (WHO) defines palliative care as the prevention and relief of suffering of adult and pediatric patients and their families facing the problems associated with a life-threatening illness [4]. These problems include physical, psychological, social and spiritual suffering of patients and psychological, social and spiritual suffering of family members [4].

Over the past two decades palliative care has developed into an essential part of mainstream medicine [5, 6]. In particular, palliative care has made remarkable progress in the field of intensive care, where ICUs were the second most common referring sites in US hospitals, accounting for 25.6% [7]. It has been shown that integrating palliative care with conventional intensive care practices can improve symptoms, improve quality of life, reduce Post Intensive Care Syndrome (PICS), and decrease costs for ICU patients [8, 9].

In Japan, however, palliative care over the last 30 years has been mainly used for cancer care, an insufficiently practiced in non-cancer diseases [10, 11]. Palliative care in the ICU in Japan was first reported in 1999 [12]. Initially it was primarily used in the pediatric field, including neonatal intensive care, then for cancer patients, but all were case reports or case series. In the 1990 and 2000s, there were several court cases in Japan regarding the withdrawal or withholding of life-sustaining treatment, and in two of these cases, the physicians were convicted of murder with a suspended sentence [13, 14]. This led to the development of guidelines for the discontinuation and withholding of life-sustaining treatment both in society and the medical community, and in 2006, JSICM issued the “Recommendation on terminal care of critically ill patients in intensive care” [15]. In 2007, Japan’s Ministry of Health, Labor and Welfare (MHLW) issued the “Guideline for the decision-making process for terminal care” [16], which outlines the basic approach to withdrawing or withholding life-sustaining treatment. The guideline can be summarized by the following four points: (1) the patient’s own wishes should be respected first; (2) if the patient’s wishes are not known, the patient’s wishes should be estimated with a surrogate decision-maker, such as a family member, and the estimated wishes should be respected; (3) if the patient’s wishes cannot be estimated, the medical team, including the patient’s family, should consider what is best for the patient, and a policy should be decided by consensus; and (4) the government needs to improve the palliative care delivery system to support patients and their families in the end of life.

In line with the principles of this national guideline, in 2014, the JSICM, the Japanese Association for Acute Medicine (JAAM) and the Japanese Circulation Society (JCS) published “Guidelines for end-of-life care in emergency and intensive care: Recommendations from three societies” [17], which triggered a full-scale movement toward palliative care in intensive care in Japan. Then, in the United States in 2013, the American College of Cardiology Foundation and American Heart Association Guidelines for the Management of Heart Failure placed palliative care for symptomatic advanced heart failure patients as a Class I (strong recommendation) recommendation [18]. In 2018, there was a major improvement in Japan’s health insurance system, and in addition to cancer, palliative care consultation for end-stage heart failure became eligible for reimbursement. As the concept of palliative care and end-of-life care continues to change, in 2021, the JCS and The Japanese Heart Failure Society issued the “Statement on palliative care in cardiovascular diseases” [19].

Thus, the concept of palliative care and end-of-life care in Japan has changed dramatically and the application of palliative care in ICU is gradually increasing. How, and to what extent, is palliative care actually being provided in the ICU clinical setting remains unknown and has not yet been investigated. This study aimed to assess the current delivery and self-reported practice of palliative care in ICUs in Japan. We also collected physician’s perspectives on the appropriate timing for the introduction of palliative care, and the discussion with ICU patients and their families on the goals of care.

## Methods

### Participants and procedure

To identify all ICUs in Japan, we first identified all 579 institutions whose ICUs calculate the specified ICU management fee set by the Japan’s MHLW [20–27]. We then included all 294 institutions registered as emergency and critical care centers by JAAM, which provide tertiary emergency medical care for severe diseases and as such, should have emergency medicine ICUs [28]. This identified 873 institutions in total. There were 209 institutions that were common to both lists.

In August 2020, we mailed the survey for a self-administered questionnaire to all 873 institutions. We requested the physician director of each ICU respond to the questionnaire, defining the physician director as a physician who can appropriately represent and answer questions regarding clinical care in the ICU. In the case of the 209 overlapping institutions, two questionnaires were sent, and if the physician director was the same person, we asked them to respond to both questionnaires from the perspective of each ICU situation, the emergency medicine ICU and the other types of ICUs. If there were multiple ICUs at the institution, where the specific ICU management fee was calculated (for example, a cardiovascular ICU and a pediatric ICU), it was left up to the institution to decide which ICU responded. Responses to the questionnaire were anonymous, and responders were not identified. A checkbox item designated “participation” and the return of a completed questionnaire was considered as consent for participation in the study. A reminder was sent out to non-respondents 1 month after the first mail. This study was conducted with the approval of the ethics committee of Tohoku University (No. 2020-1-231) and Kobe University School of Medicine (No. B200018).

### Questionnaire

The draft questionnaire was developed by the authors based on literature review [29–32], and revised following discussions with 12 multi-disciplinary specialists including palliative care physicians, intensivists, emergency physicians, nurses, and palliative care researchers. Face validity and contents validity were confirmed with 10 intensive care physicians who had worked in ICUs.

### Participant characteristics

We surveyed participant demographics including age, years since receiving a medical license, and the number of years practicing in ICUs. In addition, we asked participants about the number of beds and types of ICUs in their institution, the latter being a multiple choice question, with the following seven options: emergency medicine ICU, general ICU, medical ICU, surgical ICU, cardiac ICU, neurosurgical ICU, and other. If more than one type is applicable, the respondents were asked to select one type in which they mainly work. These questions were modified from a previous study [29].

### Structure of palliative care provision in ICU

To identify the structure of palliative care provision in ICU, we asked the following six questions, which were modified from a previous study [29]:

1. The degree of implementation of palliative care screening in ICUs on a 5-point Likert-like scale (1: always, 2: sometimes, 3: not much, 4: not at all, 5: not known). In general, palliative care screening in the ICU can be divided into two categories based on its purpose: 1) to identify patients who need palliative care and their needs, and 2) to identify patients who need to be referred to specialized palliative care. Here, we asked whether palliative care screening was being conducted for either of these purposes.
2. Which methods were used to identify patients who need palliative care in the ICU, with seven options allowing for multiple responses: acute prognosis prediction tools (such as acute physiology and chronic health evaluation II (APACHE II) or sequential organ failure assessment score (SOFA score)); end-of-life prognostic tools (such as palliative prognosis score (PaP score)); institution-specific palliative care screening tools; prediction of prognosis by clinician’s experience; discussions in the multidisciplinary conference on palliative care; no clear method was used; and other.
3. The frequency of multidisciplinary conferences on palliative care, with six options: not at all, once a month, twice a month, three times a month, four times a month, five or more times a month). In this study, a multidisciplinary palliative care conference refers to conferences on palliative care of patients attended by multidisciplinary medical professionals, including physicians.
4. The existence of standardized protocols for symptom management and care aimed to alleviate the suffering of terminally ill patients, specifically on the following 11 items allowing multiple answers: analgesia, sedation, delirium, ventilator weaning for discontinuation of life-sustaining treatment, extubation of tracheal tube for discontinuation of life-sustaining treatment, symptom relief for intractable dyspnea, symptom relief after discontinuation of life-sustaining treatment, end-of-life infusion management, discontinuation of nutritional therapy, discontinuation of drugs not required for symptom alleviation at the end of life, and other.
5. Whether the following 10 symptoms were regularly measured on an everyday basis using scales, such as the Numerical Rating Scale (NRS), Visual Analogue Scale (VAS), Behavioral Pain Scale **(**BPS), Critical-Care Pain Observation Tool (CPOT), and Richmond Agitation-Sedation Scale (RASS), allowing multiple answers: pain, delirium, dyspnea, insomnia, bowel movement, anxiety/depression, nausea, fatigue, thirst, and other.
6. Whether there was a system of psychosocial support for the family, using binary a answer (yes or no).

### The physician’s perception of the adequacy of symptom relief, provision of information and PICS prevention in ICU

With patients who may have difficulty recovering in mind, we asked participants about the appropriateness of their institution’s practice in the following four items: physical symptoms, psychological distress, providing information about the expected future course of the disease, and care for prevention of PICS; using a six-point Likert scale (1: strongly agree, 2: agree, 3: somewhat agree, 4: somewhat disagree, 5: disagree, 6: strongly disagree).

### Goals-of-care discussion

These questions were modified from previous studies [29, 30].

1. The practice of goals-of-care discussions using a four-point Likert scale (1: always, 2: usually, 3: not much, 4: not at all) on the following six items: (1) discussions are held with the family, (2) a nurse is involved; (3) using a room that ensures privacy; (4) the understanding of the patient’s family is confirmed; (5) details of the discussion are documented in the medical record; and (6) a summary of the discussion is provided to the family.
2. The frequency of goals-of-care discussions with patient families on the following nine topics using a four-point Likert scale (1: always, 2: usually, 3: sometimes, 4: not at all): estimated prognosis; expected future course of the disease; identification of surrogate decision-maker of the patient; contents of advance care planning (ACP) discussion or advance directives (AD); estimated patient’s preferred treatment; family’s preferred treatment and care; withholding or withdrawing life-sustaining treatment; preferred place of care; physical, psychological, or social problems in the family.

### The physician’s perspective on the appropriateness of timing for the introduction of palliative care and conduct of goals-of-care discussion

The appropriateness of timing to introduce palliative care, using eight options (within 24 h of ICU admission; when the ICU stay is more than 7 days; when the patient’s distress is apparent; when it is futile to continue intensive care; when death is expected in ICU; when distress is expected to persist after discharge from the hospital; at the request of the patient or family; and others). This question was modified from a previous study [31].The appropriateness of timing to conduct the goals-of-care discussion, with eight options (within 24 h of ICU admission; within 72 h of ICU admission; when the patient’s distress is apparent; when it is futile to continue intensive care; when death is expected in ICU; when distress is expected to persist after discharge from the hospital; at the request of the patient or family; and other).

### Comparison between emergency medicine ICUs and non-emergency ICUs

We compared the status of palliative care in Japanese ICUs between emergency medicine ICUs and other types of ICUs.

### The relationship between palliative care delivery and the appropriateness of palliative care practice from the physicians’ perspectives

We next examined the relationship between palliative care delivery and the proportion of facilities, where palliative care is appropriately practiced from the perspective of physicians. Palliative care delivery was defined by the following four items: conducted palliative care screening (by responding 1: always in 5-point Likert-like scale); held multidisciplinary conferences at least once a month; had at least two symptom relief protocols for terminally ill patients; and monitored more than four symptoms on a daily basis using scales. Facilities where palliative care was appropriately implemented were operationally defined as: (1) providing physical and psychological symptom relief, information and PICS prevention (by responding 1: strongly agree, 2: agree, 3: somewhat agree in 6-point Likert scale); and (2) the introduction of palliative care and the conduct of goals-of-care discussion occurred within 24 h of ICU admission.

### Analysis

All statistical analyses were performed with EZR (Saitama Medical Center, Jichi Medical University, Saitama, Japan), which is modified version of R (The R Foundation for Statistical Computing, Vienna, Austria) [33]. We used a Fischer’s exact test to assess differences between emergency medicine ICUs and non-emergency ICUs and the relationship between the palliative care delivery and the appropriateness of palliative care practice from the physicians’ perspectives. Statistical significance was assumed if *P* values were 0.05 or less.

## Results

Of the 873 institutions sent the questionnaire, 439 responded. Of these, three respondents did not answer the questionnaire to result in 436 institutions with valid responses to the questionnaire (valid response rate: 50%). Of these 436 institutions, 23 emergency and critical care centers without ICUs were excluded, resulting in 413 institutions in the analysis. Table 1 outlines the study participant backgrounds. The mean age of the respondents was 50.7 ± 8.4 years, the mean years since receiving a medical license was 25.1 ± 8.3 years, and the mean years practicing in the ICU was 14.5 ± 9.0 years.

**Table 1:** Characteristics of the participants and their institutions

		*n* = 413		
		*n*	%	(95% CI)
Age				
Mean (± SD)		50.7 (± 8.4)		
30–39 years		44	11	(8–14)
40–49 years		130	31	(27–36)
50–59 years		173	42	(37–47)
≥60 years		60	15	(11–18)
Years since receiving a medical license				
Mean (± SD)		25.2 (± 8.3)		
≤9 years		9	2	(1–4)
10–19 years		96	23	(19–28)
20–29 years		155	38	(33–42)
≥30 years		134	32	(28–37)
Years practicing in ICU				
Mean (± SD)		14.5 (± 9.0)		
≤9 years		125	30	(26–35)
10–19 years		158	38	(34–43)
20–29 years		85	21	(17–25)
≥30 years		33	8	(6–11)
Number of beds in the hospital, where the ICU is				
≤300 beds	41		10	(7–13)
301–500 beds	142		34	(30–39)
501–750 beds	140		34	(30–39)
751–1000 beds	66		16	(13–20)
≥1001 beds	20		5	(3–7)
Type of ICU				
General ICU	239		58	(53–63)
Emergency medicine ICU	122		30	(25–34)
Cardiac ICU	22		5	(4–8)
Surgical ICU	20		5	(3–7)
Medical ICU	4		1	(0–2)
Others	4		1	(0–2)
Neurosurgical ICU	0		0	(0–1)

Percentages do not add up to 100% due to missing values

*CI* confidence interval

*SD* standard deviation

*ICU* intensive care unit

### Structure of palliative care provision in ICU

Table 2 shows the results for the six questions on the structure of palliative care provision in ICU. Only 4% of the participants answered that they always provided palliative care screening for patients admitted to ICU. The common method to determine whether ICU patients were eligible for palliative care were, in order of frequency, “prediction of prognosis by clinician’s experience” (54%), “multidisciplinary conference on palliative care” (31%) and “acute prognosis prediction tools (APACHE II, SOFA score, etc.)” (21%). Only 2% of participants answered they use “their institution-specific palliative care screening tools”. In addition, 31% of participants answered there was no clear method to decide which patients need palliative care. In regards to the frequency of multidisciplinary conferences on palliative care, 75% of participants reported that conferences were never held, and only 8% answered they were held four or more times a month.

**Table 2:** Structure of palliative care provision in ICU

	*n* = 413		
	*n*	%	(95% CI)
The degree of implementation of palliative care screening in ICUs			
Yes	16	4	(2–6)
Which methods do you use to determine whether ICU patients are eligible for palliative care? (Multiple answers allowed)			
Prediction of prognosis by clinician’s experience	224	54	(50–59)
Multidisciplinary conference on palliative care	126	31	(26–35)
Acute prognosis prediction tools (APACHE II, SOFA score, etc.)	85	21	(17–25)
End-of-life prognostic tools (PaP score, etc.)	14	3	(2–6)
Your institution-specific palliative care screening tools	7	2	(1–3)
Others	6	1	(1–3)
No clear method was used	129	31	(27–36)
How often multidisciplinary conferences on palliative care are held in a month?			
Not at all	308	75	(70–79)
Once	50	12	(9–16)
Twice	10	2	(1–4)
Three times	2	0	(0–2)
Four times	24	6	(4–9)
Five or more times	8	2	(1–4)
Do you have a standardized protocol for each of the following items to alleviate the suffering of terminally ill patients? (Multiple answers allowed)			
Analgesia	112	27	(23–32)
Delirium	110	27	(23–31)
Sedation	108	26	(22–31)
Ventilator weaning for discontinuation of life-sustaining treatment	17	4	(3–6)
Discontinuation of medications not necessary for palliation of end-stage symptoms (e.g., discontinuation of hypertensive agents)	15	4	(2–6)
Extubation of tracheal tube for discontinuation of life-sustaining treatment	14	3	(2–6)
Symptom relief for difficult-to-treat respiratory failure	12	3	(2–5)
End-of-life infusion management	9	2	(1–4)
Discontinuation of nutritional therapy	7	2	(1–3)
Symptom relief after discontinuation of life-sustaining treatment	4	1	(0–2)
Others	24	6	(4–9)
Are you continuously assessing for each of the following symptoms? (Multiple answers allowed)			
Pain	382	92	(90–95)
Delirium	340	82	(78–86)
Dyspnea	169	41	(36–46)
Insomnia	164	40	(35–45)
Bowel movement (Constipation/Diarrhea))	142	34	(30–39)
Anxiety/Depression	133	32	(28–37)
Nausea	104	25	(21–30)
Fatigue	37	9	(7–12)
Thirst	29	7	(5–10)
Others	8	2	(1–4)
Do you have a system of psychosocial support for the family? (e.g., interviews with a clinical psychologist, information on social resources provided by a social worker)			
Yes^a^	230	56	(51–60)

Percentages do not add up to 100% due to missing values or duplicate responses

ICU, intensive care unit

CI, confidence interval

The number and percentage who answered ‘always’ on a 5-point Likert scale for the question was described

APACHE II, Acute Physiology and Chronic Health Evaluation

SOFA score, Sequential Organ Failure Assessment score

PAP score, Palliative Prognosis Score

^a^The number and percentage who answered ‘yes’

According to participant responses, the prevalence of standardized protocols for symptom relief and other measures aimed at alleviating the suffering of terminally ill patients were, in descending order, analgesia (27%), sedation (27%), and delirium (26%). Other standardized protocols such as discontinuing treatment such as ventilator weaning (4%), discontinuation of medications not necessary for the palliation of end-stage symptoms (4%), and extubation of tracheal tube (3%), were much less common. Fifty-four percent of respondents answered that they had no standardized protocols.

The frequency of symptoms regularly measured on a daily basis using scales such as NRS, VAS, BPS, CPOT and RASS were, in descending order, pain (92%), delirium (82%), dyspnea (41%), insomnia (40%), bowel movement (constipation/diarrhea)) (34%), anxiety/depression (32%), and nausea (25%). A system of psychosocial support for the family was present according to 56% of participants.

### The physician’s perception of the adequacy of symptom relief, provision of information and PICS prevention in ICU

**Fig. 1:**
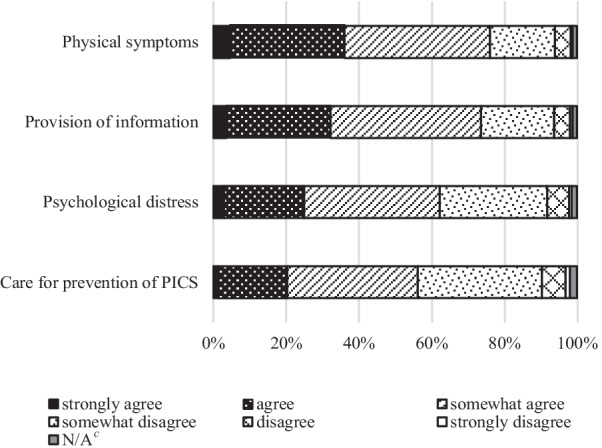
Physician’s perception of appropriateness of symptom relief, provision of information and PICS^a^ prevention in ICU^b^. ^a^*PICS* post intensive care syndrome, ^b^ICU intensive care unit, ^c^N/A not answered

Participants were asked questions relating to patients who may have difficulty recovering. As shown in Fig. 1, items where physicians thought they provided appropriate palliative care (answered “strongly agree” and “agree”) were, in decreasing order of frequency, 36% (95% CI 32–41) in physical symptoms, 32% (95% CI: 28–37) in the provision of information, 25% (95% CI: 21–29) in psychological distress and 20% (95% CI: 17–24) in PICS prevention.

### Goals-of-care discussions

Table 3 shows how participants provide goals-of-care discussions in ICU. More than 80% of participants indicated they document details of the discussion in the medical record (96%), ensure privacy (90%), involve a nurse with the discussion (87%), and ensure the understanding of the patient’s family (85%). While 82% had discussions with the families, only 58% of participants indicated they gave a summary of the discussion to the family.

**Table 3:** Structure of goals-of-care discussions in ICU

	*n* = 413		
	*n*	%	(95% CI)
Details of the discussion are documented in the medical record^a^	397	96	(94–98)
Using a room that ensures privacy^a^	372	90	(87–93)
A nurse is involved^a^	361	87	(84–90)
The understanding of the patient’s family is confirmed^a^	353	85	(82–89)
Discussions are held with the family^a^	340	82	(78–86)
A summary of the discussion is provided to the family^a^	240	58	(53–63)

Percentages do not add up to 100% due to duplicate responses

*ICU* intensive care unit

*CI* confidence interval

^a^The number and percentage who answered ‘always’ and ‘usually’ on a 4-point Likert scale

The contents discussed in the goals-of-care discussions are shown in Table 4. Commonly discussed contents were, in descending order, “expected future course of the disease” (97%), “estimated prognosis” (95%), “family’s preferred treatment and care” (92%), “estimated patient’s preferred treatment” (86%), and “withholding or withdrawing life-sustaining treatment” (79%). In contrast, “physical, psychological, or social problems in the family” (68%) and “contents of ACP discussion or AD” (65%) were relatively less frequently discussed.

**Table 4:** Frequency of contents of goals-of-care discussion with patient’s family in ICU

	*n* = 413		
	*n*	%	(95% CI)
Expected future course of the disease^a^	401	97	(95–98)
Estimated prognosis^a^	392	95	(92–97)
Family’s preferred treatment and care^a^	382	92	(90–95)
Estimated patient’s preferred treatment^a^	354	86	(82–89)
Withholding or withdrawing life sustaining treatment^a^	325	79	(74–82)
Identification of surrogate decision-maker of the patient^a^	323	78	(74–82)
Preferred place of care^a^	294	71	(67–75)
Physical, psychological, or social problems in the family^a^	279	68	(63–72)
Contents of advance care planning discussion or advance directives^a^	267	65	(60–69)

Percentages do not add up to 100% due to duplicate responses

ICU intensive care unit

CI confidence interval

^a^The number and percentage who answered ‘always’ and ‘usually’ on a 4-point Likert scale

### Appropriate timing for the introduction of palliative care and for conducting goals-of-care discussion from the physician’s perspective

The most commonly responses in regards to what physicians considered the most appropriate timing for the introduction of palliative care are shown in Table 5. They were, in descending order, “when it is futile to continue intensive care” (68%), “at the request of the patient or family” (55%), “when the patient’s distress is apparent” (53%), “when death is expected in ICU” (52%), “when distress is expected to persist after discharge from the hospital” (34%), “within 24 h of ICU admission” (15%), and “when the ICU stay is more than seven days” (13%).

**Table 5:** Appropriate timing for the introduction of palliative care and conducting goals-of-care discussions (physician’s perspective)

	*n* = 413		
	*n*	%	(95% CI)
When do you think it is appropriate timing to introduce palliative care? (Multiple answers allowed)			
When it is futile to continue intensive care	279	68	(63–72)
At the request of the patient or family	228	55	(50–60)
When the patient’s distress is apparent	217	53	(48–57)
When death is expected in ICU	213	52	(47–56)
When distress is expected to persist after discharge from the hospital	142	34	(30–39)
Within 24 h of ICU admission	60	15	(11–18)
When the ICU stay is more than 7 days	54	13	(10–17)
Others	12	3	(2–5)
When do you think it would be appropriate to conduct goals-of-care discussion? (Multiple answers allowed)			
When it is futile to continue intensive care	293	71	(66–75)
When death is expected in ICU	268	65	(60–69)
At the request of the patient or family	241	58	(54–63)
When the patient’s distress is apparent	168	41	(36–45)
Within 24 h of ICU admission	166	40	(36–45)
When distress is expected to persist after discharge from the hospital	99	24	(20–28)
Within 72 h of ICU admission	85	21	(17–25)
Others	9	2	(1–4)

Percentages do not add up to 100% due to duplicate responses

*CI* confidence interval

*ICU* intensive care unit

Responses to the most appropriate timing for conducting the goals-of-care discussion were, in descending order, “when it is futile to continue intensive care” (71%), “when death is expected in ICU” (65%), “at the request of the patient or family” (58%), “when the patient’s distress is apparent” (41%), “within 24 h of ICU admission” (40%), “when distress is expected to persist after discharge from the hospital” (24%), and “within 72 h of ICU admission” (21%).

### Comparison between emergency medicine ICUs and non-emergency ICUs

This comparison was performed to determine if there were any differences between emergency medicine ICUs and other types of ICUs. There was no significant difference except for responses to the most appropriate timing for conducting the goals-of-care discussion. A higher percentage of directors of emergency medicine ICUs answered that it would be appropriate to have the discussion “within 24 h of ICU admission” than directors of other types of ICU (54% vs 34%, *P* value = 0.019).

### The relationship between palliative care delivery and the appropriateness of palliative care practice from the physicians’ perspectives

In ICUs where palliative care screening was conducted, a significantly higher percentage of respondents answered that palliation of physical symptoms was provided adequately (100 vs. 76%, *P*-value = 0.029). In ICUs where multidisciplinary conferences were held at least once a month, a significantly higher percentage of respondents answered “within 24 h of ICU admission” as the appropriate timing to introduce palliative care (23 vs. 12%, *P*-value = 0.023). In ICUs with two or more standardized protocols for symptom management and care for alleviation of suffering in terminally ill patients, a significantly higher percentage of respondents reported that palliation of physical symptoms (90 vs. 71%, *P*-value < 0.001), psychological distress (83 vs. 54%, *P*-value < 0.001), providing information about the expected future course of the disease (95 vs. 65%, *P*-value < 0.001), and care for prevention of PICS (76 vs. 49%, *P*-value < 0.001) were provided adequately. In ICUs regularly measuring at least five symptoms on an everyday basis using scales, a significantly higher percentage of respondents answered that palliation of physical symptoms was adequate (83 vs. 74%, *P*-value = 0.042).

## Discussion

This study is the first study to clarify the current status of palliative care practices in ICUs in Japan, including palliative care screening and symptom control from the perspective of the physician director.

We identified six key findings in this study. The first and most important finding was that only 20–36% of ICU physicians believed palliative care, including physical and psychological symptom management, communication, and PICS prevention, was adequately provided. This self-assessment of palliative care practice is difficult to evaluate correctly, because there are no absolute standards and no similar previous studies, but it is a low figure based on the current understanding of the benefits of palliative care. Furthermore, as many patients in ICU are under sedation for reasons such as mechanical ventilation, the degree of their distressing symptoms may be underestimated. These results indicate that more focus on improving symptom relief is needed in the ICU, such as educating ICU physicians on symptom management and palliative care.

The second important finding of this study was that very few ICUs implemented palliative care screening, and many ICUs had no criteria for determining which patients required palliative care. End-of-life care for dying patients is only one component of palliative care in ICU, it is also important to care for patients and their families who are uncertain about their recovery from intensive care [34]. However, the results of this study indicate that more than half the time, the timing of the introduction of palliative care is determined by the empirical judgment of physicians, and more than half of the physicians responded that the appropriate timing was when treatment became futile. This indicates that the introduction of palliative care by physicians is often late and inappropriate. It suggests the essential understanding of palliative care may be insufficient, and palliative care is being used only as end-of-life care. Specifically, at the initiation of treatment in the ICU, the focus may be solely on prolonging the patient’s life, without paying attention to uncertainty of the patient’s prognosis and of meaningful functional recovery. It is likely that only once survival seems impossible and curative management is gradually withheld that palliative care is provided, at this point, possibly inadequately. In addition, in our study, from the physician’s perspective, facilities that implemented palliative care screening were more likely to have adequate palliation of physical symptoms.

To improve the quality of life of patients and their families, screening may be an important solution to acknowledge their unmet palliative care needs, with proper recognition of the uncertainty of patient recovery. It has been reported that the early introduction of palliative care reduced medical costs in the ICU, increased hospice transfers, decreased post-discharge emergency room visits, and decreased post-discharge readmissions without reducing in-hospital mortality or 30-day mortality [7]. Integration of intensive care and palliative care in the ICU could be an important factor in improving the quality of life of patients and their families.

Our third finding was that even though pain, sedation and delirium are continuously monitored in the ICU, only about a quarter of facilities were found to have symptom relief protocols for these specific symptoms. This is consistent with a previous study in Italy (30%) [29]. In addition to that, our study found facilities with standardized protocols for symptom management for terminally ill patients provide a more appropriate palliation of physical and psychological symptoms and PICS prevention. These results indicate that these symptoms have been identified as items requiring symptom management and show there is an opportunity to improve symptom control with the implementation of standardized management practices.

Our fourth finding was in most ICUs there were no standardized protocols related to the withdrawal of life-sustaining treatment, or symptom management after withdrawal of life-sustaining treatment. Internationally, extubation is an important procedure in ICU palliative care [35–37], and when extubation is deemed appropriate, it is recommended that ventilation be discontinued as quickly as possible, with the patient’s comfort being the highest priority^38^. In Japan, there are guidelines for the decision-making process regarding the withholding and withdrawal of life-sustaining treatment [39]. While it is possible to withdraw treatment, the lack of clear criteria and laws regarding physician exemption may be the reason for our finding of a lack of standardized protocols. Several court decisions have ruled that life-sustaining treatment can be discontinued if certain conditions are met [13, 14]. These conditions are: (1) limitation of the duty to treat: the patient is suffering from an untreatable disease and is in a terminal condition with no hope of recovery and death is inevitable (repeated diagnosis by multiple doctors is desirable); and (2) the right of the patient to self-determination: the patient’s expressed will to discontinue the treatment must exist at the time of discontinuation, and if there is no clear expression of will at the stage of considering discontinuation, the patient’s presumptive will should be recognized. In addition, according to “The national survey on attitudes toward medical care in the final stages of life”, only 28.6% of physicians refer to the guidelines, and their dissemination is not sufficient [40]. Lack of dissemination and understanding of these laws and guidelines to both medical professionals and the public may be one of the major barriers to the widespread use of withdrawal of life-sustaining treatment and its protocols. In the future, it may be necessary to educate medical professionals on the knowledge of withholding or withdrawing of life-sustaining treatment, and to develop standardized protocols for them including terminal weaning and extubation, while also obtaining consensus from academic societies and the general public.

The fifth important finding is that 74% of the facilities did not hold multidisciplinary conferences on palliative care. A multidisciplinary approach is considered to be an important role in ICU palliative care [34]. Our finding that regular multidisciplinary conferences on palliative care are rarely held in ICUs may be one manifestation of the lack of integration of palliative care into ICU care in Japan. Our study found that in ICUs that hold multidisciplinary conferences at least once a month, a significantly higher percentage of respondents considered the appropriate timing to introduce palliative care was within 24 h of ICU admission. By conducting multidisciplinary conferences, there is a possibility that the comprehensive needs of patients and their families can be recognized within the team and care can be improved. Palliative care needs to be developed and integrated into intensive care by introducing both palliative care screening and multidisciplinary conferences.

The sixth important finding was that although goals-of-care discussions with family members were held, the content of ACP and AD and the psychosocial issues of family members were not adequately discussed. The ACP process itself and the ADs left as a result of the ACP discussions has the potential to (1) allow patients, those close to them and clinical teams to better utilize shared decision making when planning care; (2) reduce confusion and conflict when patients are acutely ill, have lost capacity and have a high risk of dying; (3) improve the clarity of communication surrounding care at the end of life and reduce the severity of grief amongst friends and families; and (4) reduce the incidence and impact of burn-out in healthcare professionals [41]. As part of the initial assessment of palliative care, it is considered best practice to identify a pre-existing AD or contents of ACP discussions at the time of admission or within 24 h of admission [42, 43]. The previously mentioned Japanese national survey on attitudes toward medical care in the final stages of life found 75.5% of the general public and 41.6% of physicians had never heard of ACP. This increased to 94.7 and 76.1%, respectively, when also including those who responded”I have heard of it but do not know much about it”[40]. In addition, the same survey found the rate of ACP practice by physicians was also low at 27.3%, which may be due to the insufficient dissemination of the concept of ACP in Japan.

In addition, the following two factors may act as barriers to disseminating palliative care in ICUs in Japan. First is the high-cost medical expense system currently in place, which sets a limit (40,000–200,000 yen) on medical expenses based on income, and monthly payments above the limit are refunded at a later date. Since patients, their families, and medical professionals can continue treatment and care, including life-sustaining treatment without incurring costs, the introduction of primary palliative care tends to be postponed. The other barrier relates to how the health insurance system reimburses specialized palliative care. In Japan, inpatient hospice/palliative care units are only available for patients with cancer and acquired immunodeficiency syndrome. In addition, palliative care consultations cannot be reimbursed except for patients with cancer or end-stage heart failure. This may limit referrals to specialized palliative care.

### Implications to practice

It is evident from this study that the integration of palliative care and ICU care is still inadequate in Japan. The results of this study should be used consider how best to screen all patients admitted to the ICU, conduct multidisciplinary conferences for patients who have screened positive, and identify solutions for these patients. This may help identify the potential comprehensive palliative care needs of ICU patients and their families and to respond appropriately to those needs.

Another suggestion stemming from this study is to develop a standardized protocol for symptom management and the withholding or withdrawing of treatment. In addition to improving symptom relief for patients it would also reduce the legal risks for healthcare providers. Furthermore, the development of a pathway for treatment discontinuation may lower the barrier to active treatment. In Japan, patients and their families may be reluctant to start endotracheal intubation and mechanical ventilation if they are told that extubation is not possible once these two procedures have started [44]. The inability to withdraw life-sustaining treatment if unsuccessful may make a patient hesitant to choose active treatment. Creating and disseminating a standardized protocol for treatment withdrawal driven by academic societies with the patient and public involvement would help improve this situation.

In addition to this, to promote appropriate referral to specialized palliative care and integration of intensive care and palliative care, it may be effective to have palliative care consults covered by health insurance for all diseases, not just cancer and end-stage heart failure.

### Limitations

This study had several limitations. First, the response rate was 50%, which is not a low response rate for a survey of doctors, but the results may not accurately reflect the current situation of palliative care in ICUs in Japan. Second, if an institution had multiple ICUs, we let respondents decide which ICU to consider when answering the questionnaire. Therefore, the results of this study may not reflect the actual situation in all ICUs. Thirdly, respondents may not always be familiar with the reality of palliative care in daily ICU practice. The results of this study may be better interpreted in conjunction with the results of an ongoing national survey of nurses in Japan. Fourth, the results for appropriate symptom relief, explanation of illness, and goals-of-care discussion are based on physicians’ overall evaluations and may not reflect the actual state of treatment and care for patients and their families. Prospective observational studies and surveys of bereaved families should be conducted to more accurately evaluate the situation. Finally, the questionnaire used in this study has not been examined for reliability or validity.

## Conclusions

Through this survey, it became clear that palliative care is not sufficiently implemented in Japanese ICUs and that the integration of palliative care and intensive care is far from complete. For further dissemination and integration of palliative care in the ICU, it is important to identify unmet needs through palliative care screening, conduct multidisciplinary conferences to find adequate solutions for these unmet needs, develop standardized protocols including symptom management and withholding or withdrawing of life-sustaining treatment, and educate intensivists on these procedures.

## Data Availability

The data sets used and/or analyzed during the current study are available from the corresponding author on reasonable request.

## Abbreviations

ICU: Intensive care units
CI: Confidence interval
JIPAD: Japanese Intensive Care PAtient Database
JSICM: The Japanese Society of Intensive Care Medicine
WHO: World Health Organization
PICS: Post Intensive Care Syndrome
MHLW: Ministry of Health, Labor and Welfare
JAAM: The Japanese Association for Acute Medicine
JCS: The Japanese Circulation Society
APACHE II: Acute physiology and chronic health evaluation II
SOFA score: Sequential organ failure assessment score
PaP score: Palliative prognosis score
NRS: Numerical Rating Scale
VAS: Visual Analogue Scale
BPS: Behavioral Pain Scale
CPOT: Critical-Care Pain Observation Tool
RASS: Richmond Agitation–Sedation Scale
ACP: Advance care planning
AD: Advance directives
